# Tuning Thermochemistry Behavior of Coal Gasification Fine Ash via Alkyl Chain-Length-Dependent Surface Functionalization: Mechanisms and Structure–Property Relationships

**DOI:** 10.3390/molecules31101682

**Published:** 2026-05-15

**Authors:** Luzhen Jiao, Huiguo Yu, Yanshun Li, Yiqun Chen, Jiawei Li, Xiaoguang Li

**Affiliations:** 1 Department of Biological and Chemical Engineering, Shandong Vocational College of Science and Technology, 6388 West Ring Road, Weifang 261053, China; 2 School of Energy and Power Engineering, Northeast Electric Power University, 169 Changchun Street, Jilin 132012, China; 3 School of Energy and Environmental Engineering, University of Science and Technology Beijing, 30 Xueyuan Road, Beijing 100083, China

**Keywords:** CGFA, alkylation reaction, structure characterization, thermal conversion, chemical modification

## Abstract

Coal gasification fine ash (CGFA) is a carbon–mineral composite solid waste whose valorization is severely hindered by poor interfacial compatibility with organic media due to its highly polar surface. Here, we report a surface alkylation strategy using haloalkanes with variable chain lengths to systematically tune the surface chemistry and thermo-oxidative behavior of CGFA. Comprehensive spectroscopic characterizations (XPS, FTIR, and ^13^C NMR) confirm successful grafting of alkyl chains, which increases aliphatic C-H content from 24.8% to 43.9% while reducing polar carboxyl groups from 7.9% to 1.6%, with the mineral framework remaining intact. Thermogravimetric analysis reveals that alkylation lowers the onset decomposition temperature from 358 °C to 295 °C and enhances the maximum mass-loss rate. Kinetic analysis shows that grafted alkyl chains act as low-energy initiation sites, reducing the initial activation energy to 95 kJ/mol, while the later-stage oxidation becomes diffusion-limited. Notably, long straight-chain alkylation achieves the best performance, whereas branched chains are less effective due to steric hindrance and pore blockage. This work establishes a clear chain-length-dependent structure–thermal response relationship, positioning alkylated CGFA as a designable precursor for functional carbon materials, intelligent char-forming agents, and tunable components for energy or responsive material systems.

## 1. Introduction

Coal gasification is a crucial technology for the clean and efficient conversion of coal, widely employed in energy supply and chemical feedstock production. However, this process inevitably generates substantial amounts of solid byproduct [1,2]. Among them, coal gasification fine ash (CGFA) possesses more complex physicochemical characteristics due to its fine particle size, significant compositional fluctuations from varying feedstocks and operating conditions, and the tight coupling between its carbonaceous and mineral phases [3]. The physicochemical properties of CGFA are highly dependent on the coal gasification technology employed. Entrained-flow gasification (typically 1200–1600 °C) produces fine ash with lower carbon content and more fully fused mineral phases, while fluidized-bed gasification (800–1000 °C) generates ash with higher residual carbon and less crystallized mineral matter. Compared to conventional coal combustion fly ash, CGFA typically exhibits a more pronounced multiphase composite nature, higher surface chemical heterogeneity, and coexisting multiscale pore structures [4]. These attributes collectively lead to common bottlenecks in its valorization: difficult separation, poor stability, and poor matching to application scenarios [5,6]. Without targeted regulation of its structure and surface properties, CGFA often ends up in low-value disposal routes, resulting in resource wastage and environmental burdens [7].

From a materials application perspective, CGFA is attractive as a low-cost carbon–mineral composite micropowder with functional potential [8,9]. Its inherent structure suggests promise for applications such as a precursor for functional carbon materials via controlled pyrolysis, a flame-retardant synergist or char-forming agent in polymer composites, a precursor for energy storage materials, and a component in stimuli-responsive material systems [10,11]. In these scenarios spanning polymers, rubbers, coatings, asphalt, or media involving organic phases, CGFA could serve as a functional particulate component [12], provided that stable dispersion and good interfacial compatibility are achieved [13,14]. Nevertheless, pristine CGFA commonly contains diverse oxygen-containing functional groups and inorganic ash components [15], leading to complex surface energy and polarity distributions. This makes it prone to moisture uptake and agglomeration [16], while resulting in poor wettability and dispersion in nonpolar or weakly polar media, severely limiting its application in organic systems [17]. A central contradiction thus exists: CGFA possesses both resource attributes and structural potential, yet insufficient interfacial compatibility and dispersion stability hinder its transition from a solid waste to a functional material [18,19].

Organic surface functionalization is an effective strategy for tuning the interfacial properties of powdered materials [20]. Since wettability, dispersibility, and interfacial compatibility are governed primarily by the outermost surface chemistry [21], such modifications often require only low grafting densities or small amounts of reagent [22]. Even at low coverage, they can significantly alter surface energy and interfacial interactions, thereby effectively regulating material behavior while largely preserving the bulk framework and mineral-phase structure [23,24]. Specifically, alkylation, by introducing hydrophobic organic chains, can reduce surface polarity and modulate surface energy and wettability, potentially improving dispersion and interfacial interactions in organic media [25]. For multiphase systems like CGFA, where carbonaceous and mineral phases coexist, the introduction of alkyl chains may not only modify surface hydrophilicity but also, through surface coverage and steric effects, influence the exposure of active sites, the chemical states of functional groups, local defect structures, and pore accessibility [26]. Notably, variations in alkyl chain-length can lead to differences in hydrophobicity, chain flexibility, and surface coverage density, thereby inducing distinct interfacial states and structural responses [27,28]. However, systematic studies on the alkylation functionalization of CGFA remain scarce. Specifically, previous works have not clarified how the length and structure of alkyl chains regulate the thermo-oxidative kinetics of CGFA or established a quantitative correlation between alkyl chain properties and the material’s thermal response behavior. The coupled relationships among alkyl chain length surface chemistry–carbon structure interfacial behavior have not been fully elucidated, particularly lacking an evidence chain that connects multidimensional characterization to quantifiable performance responses.

To bridge this knowledge gap, thermogravimetric analysis (TGA) under an oxidative atmosphere can serve as a powerful diagnostic tool, linking structural evolution to thermal response behavior. The thermo-oxidative response of carbonaceous solids is highly sensitive to defect structures, oxygen-containing functional groups, and the mineral carbon interfacial state. Consequently, the mass-loss profiles and apparent kinetic parameters derived from TGA can reflect changes in active sites, functional group rearrangements, the evolution of aromatic ordering, and catalytic or inhibitory effects associated with mineral components. Integrating TGA-based kinetics with multi-spectroscopic and multiscale structural characterization helps establish a surface functionalization–structure/interfacial state–oxidation response framework. This approach provides comparable baseline data and mechanistic insights for evaluating processing windows, stability under thermal exposure, and the selection of suitable application scenarios.

Accordingly, this study employs CGFA as the target material and performs surface alkylation functionalization using haloalkane reagents with different alkyl chain lengths, constructing a chain-length-tunable series. A combination of morphological/porosity analyses and multiple spectroscopic techniques is used to systematically reveal the changes in morphology and aggregation state, pore structure, surface elemental composition and functional group chemical states, carbon structural ordering and defect features, and crystalline phase composition induced by the modification. Oxidative atmosphere TGA is further conducted, and the Coats–Redfern method is applied to obtain apparent kinetic parameters, enabling a quantitative comparison of the oxidation response across the chain-length series through an integrated analysis of chain-length-tunable surface alkylation and structure characterization kinetic response.

The rationale for alkylating CGFA is twofold. First, pristine CGFA inherently contains abundant polar oxygen-containing groups (e.g., carboxyl, hydroxyl) on its surface, which render it hydrophilic and prone to agglomeration, thereby causing poor dispersion and interfacial incompatibility when incorporated into nonpolar or weakly polar organic matrices such as polymers, rubbers, or asphalt. Alkylation replaces these polar groups with hydrophobic aliphatic chains, fundamentally reducing surface polarity and enhancing the affinity of CGFA toward organic media. Second, the grafted alkyl chains are designed to act as molecular “fuses” that can preferentially oxidize at lower temperatures, providing a tunable handle to control the thermo-oxidative behavior of CGFA. This feature is particularly valuable for applications requiring precisely timed thermal decomposition, such as controlled pyrolysis for porous carbon production, char-forming flame retardants in polymer composites, and thermally responsive material systems.

This work aims to clarify the regulatory mechanism of alkyl chain length on the physicochemical structure and interfacial state of CGFA, and to establish an interpretable structure oxidation response relationship. The findings are expected to provide theoretical and data support for the application of CGFA in organic-matrix composite fillers, interfacial regulation, and related materials-oriented fields.

## 2. Results and Discussion

### 2.1. Ultimate Analyses Results

The alkylation modification induced regular changes in the elemental composition of CGFA. As shown in Table 1, the ultimate analysis results of the raw CGFA and alkylated samples revealed significant fluctuations in C and H contents after alkylation compared to CGFA.

The 2-CGFA modified by the long-chain alkyl reagent 1-bromoheptane showed that the highest C content is 28.97% and H content is 1.32%. Overall, the H content increased by 80–164%, directly confirming the successful grafting of alkyl chains. The fluctuations in C content were related to the carbon chain length and branched structure of the alkyl reagents: straight-chain alkanes increased carbon content due to high grafting efficiency, whereas branched structures inhibited the reaction, indicating that alkylation altered the organic composition of the fine ash by introducing aliphatic groups. The introduction of substantial C and H through alkylation enhanced the thermal conversion of coal gasification fine ash, improved its calorific value, reduced activation energy, optimized surface functional groups, and promoted resource utilization. These changes originate from the hydrophobic alkyl chains grafted onto the CGFA surface, which regulate surface chemistry, introduce low-energy reaction sites, and improve structural disorder. Such modifications collectively facilitate thermal oxidation, lower reaction energy barriers, and enhance the applicability of CGFA as a functional particulate in material systems.

### 2.2. Physical and Chemical Structure Characterization

#### 2.2.1. Pore Structure and Scanning Electron Microscopy

**Figure 1:**
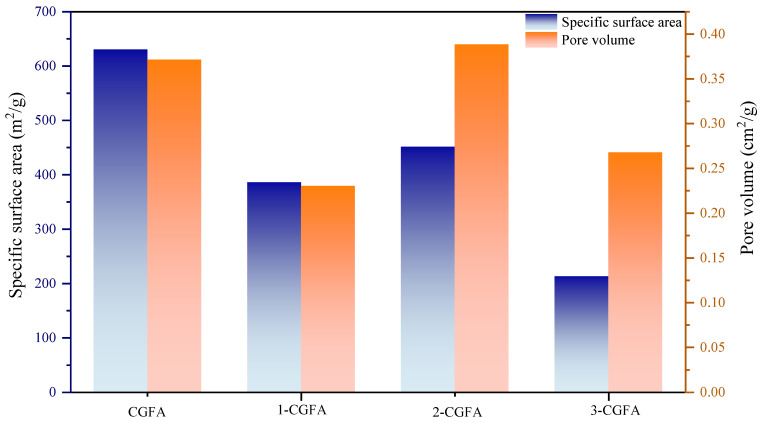
Changes in specific surface area and pore volume and after CGFA modification.

The BET surface area and pore volume measurements are displayed in Figure 1, Figures S1 and S2, showing significant changes in surface properties of CGFA samples after alkylation treatment. It can be seen that the specific surface area and pore volume of the CGFA series samples exhibit structural dependence: the initial specific surface area of the original CGFA is about 620 m^2^/g and the pore volume is about 0.39 cm^2^/g, both of which are relatively high; the 1-CGFA modified by the short chain decreased significantly to the lowest in the series, about 380 m^2^/g and 0.26 cm^2^/g. The 2-CGFA modified by the long chain achieved significant recovery of pore structure, with a specific surface area of 450 m^2^/g and a pore volume of 0.40 cm^2^/g; subsequently, the specific surface area of 3-CGFA modified with branched and long branched chains continued to decrease to 280 m^2^/g and 200 m^2^/g, respectively, but the pore volume remained relatively stable in the range of 0.28–0.38 cm^2^/g. The counterintuitive observation that long-chain alkylation (2-CGFA) causes less pore deterioration than short-chain modification (1-CGFA) can be explained by their different penetration behaviors. Short chains readily diffuse into narrow micropores and pack densely inside, blocking pore entrances and drastically reducing both surface area and pore volume.

In contrast, long straight chains adopt an extended brush-like conformation on the external surface instead of penetrating into narrow pores. This surface-localized grafting preserves the internal pore network, as evidenced by the maintained pore volume. Thus, short chains act as pore blockers while long chains serve as “surface modifiers”.

The substantial decrease in specific surface area for the short-chain alkylated sample from 620 to 380 m^2^/g can be attributed to the ability of short alkyl chains to penetrate narrow micropores and undergo dense packing, thereby blocking key gas-adsorption pathways. In contrast, despite the larger molecular weight of the long-chain alkylating reagent, its alkyl chains adopt a more extended conformation in solution. This conformation preferentially drives the grafting reaction onto the external surfaces and the openings of larger pores rather than into the internal micropore network. The grafted long alkyl chains then act as steric spacers that effectively disrupt the tight carbon–mineral agglomerates inherent to pristine CGFA, re-exposing previously encapsulated pores. Consequently, 2-CGFA maintains a relatively high specific surface area and achieves the largest pore volume among the modified samples, striking a favorable balance between surface hydrophobization and the preservation of mass-transfer channels.

**Figure 2:**
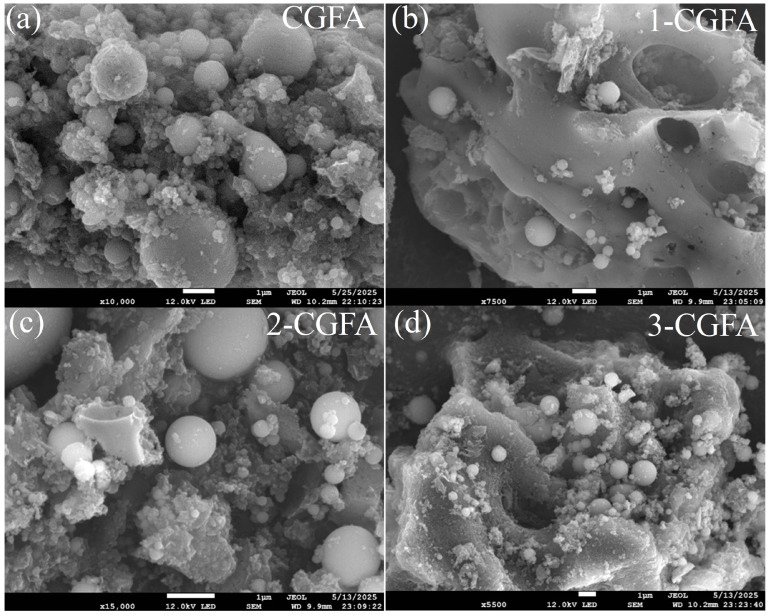
SEM images of CGFA before and after modification: (**a**) CGFA (**b**) 1-CGFA (**c**) 2-CGFA (**d**) 3-CGFA.

Figure 2 and Figure S3 show the SEM images of coal gasification fine ash before and after modification. The original CGFA surface is rough with tightly packed particles, showing clear aggregation and irregularities, indicating poor dispersion. With 1-CGFA, the surface becomes relatively smoother with reduced particle aggregation, but some aggregation still remains. With the 2-CGFA, the surface smoothness further improves, and aggregation is noticeably reduced, showing better dispersion. The surface is more uniform and 3-CGFA, with almost no aggregation, indicates excellent dispersion. The SEM images show that with increasing alkyl chain length, the surface of CGFA became smoother and the particle dispersion greatly improved. Particularly, 4-CGFA showed almost no aggregation, indicating that alkylation treatment significantly enhanced dispersion.

#### 2.2.2. XPS Result Analysis

**Figure 3:**
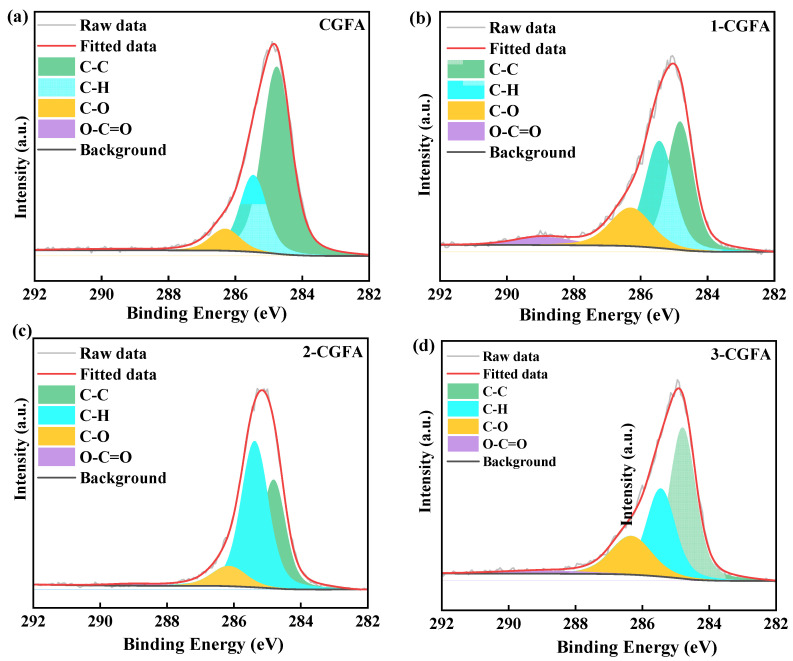
XPS spectra of CGFA and alkylation modification: (**a**) CGFA, (**b**) 1-CGFA, (**c**) 2-CGFA, (**d**) 3-CGFA.

As shown in Figure 3 and Figure S4, the high-resolution C1s spectra of CGFA and the modified samples can be deconvoluted into four carbon environments. Compared with pristine CGFA, alkylation leads to a pronounced redistribution of surface carbon species, characterized by an increase in hydrocarbon-related components, especially C-H, and a decrease in strongly polar carboxyl-type species COO^−^, indicating successful introduction and surface exposure of alkyl moieties accompanied by changes in oxygen-bonded carbon environments.

The quantitative results are summarized in Table 2. For pristine CGFA, the relative contents of C-C, C-H, C-O, and COO^−^ are 65.53%, 24.84%, 1.70%, and 7.93%, respectively. After alkylation, the C–H fraction increases overall, reaching 30.33% (1-CGFA), 43.91% (2-CGFA), and 37.69% (3-CGFA). Notably, 2-CGFA exhibits the highest C-H content of 43.91%, corresponding to an increase of 19.07 percentage points relative to CGFA, suggesting the greatest surface exposure of aliphatic carbon under this condition. Meanwhile, the C–C component decreases from 65.53% to 47.22% (1-CGFA), 45.05% (2-CGFA) and 40.24% (3-CGFA), a reduction of 17.19–25.29 percentage points, implying that the surface carbon environment shifts from being dominated by the original carbon framework to a mixed surface layer enriched with alkyl and oxygen-containing carbon.

Consistent with the bonding features expected from alkylation, the C-O contribution increases markedly from 1.70% (CGFA) to 18.96% (1-CGFA), 9.47% (2-CGFA) and 18.59% (3-CGFA), representing increases of 7.77% to 17.26%. This increase indicates a substantial enrichment of oxygen-bonded carbon environments on the surface, which is commonly consistent with the presence of C-O bonding environments after surface substitution/grafting. In contrast, the COO^−^ fraction decreases overall, from 7.93% to 3.49% (1-CGFA), 1.58% (2-CGFA), and 5.47% (3-CGFA). The lowest value is observed for 2-CGFA (1.58%), corresponding to a decrease of 6.35 percentage points. This suggests that carboxyl-type polar sites may be consumed/transformed during modification and/or become less visible to XPS due to coverage by hydrophobic chains, which is favorable for reducing surface polarity.

Importantly, the functional group distribution shows a non-monotonic trend across the series. For example, 2-CGFA simultaneously exhibits the highest C–H (43.91%) and the lowest COO^−^ (1.58%), and its total oxidized-carbon fraction is 11.05%, much lower than that of 1-CGFA (22.45%) and 3-CGFA (24.06%). This indicates that different alkylation reagents’ chain structures influence not only the extent of modification but also the surface coverage configuration and the apparent exposure of alkyl versus polar species. Overall, the XPS results provide direct surface-sensitive evidence that alkylation reconstructs the outermost functional group distribution, forming a chemical basis for interpreting subsequent differences in wettability dispersion behavior and thermo-oxidative response. The increased aliphatic carbon fraction observed by XPS is quantitatively consistent with the elevated carbon and hydrogen contents determined by elemental analysis (Table 2). The simultaneous rise in C–H moieties and overall carbon concentration confirms that alkyl chains have been successfully grafted rather than arising from surface contamination. The variation in surface isoelectric point (IEP) induced by alkylation can alter surface charge distribution and protonation states of oxygen-containing groups, thereby affecting the formation and detection of C–O bonds during grafting. Alkyl chains reduce surface polarity and shift the IEP, weakening electrostatic interactions and promoting the substitution reaction between alkylating agents and oxygenated sites. This further stabilizes the C–O bonding structure and contributes to the enhanced C–O component observed in the XPS results, confirming the coupling effect of surface charge and chemical bonding.

#### 2.2.3. FTIR Analysis

**Figure 4:**
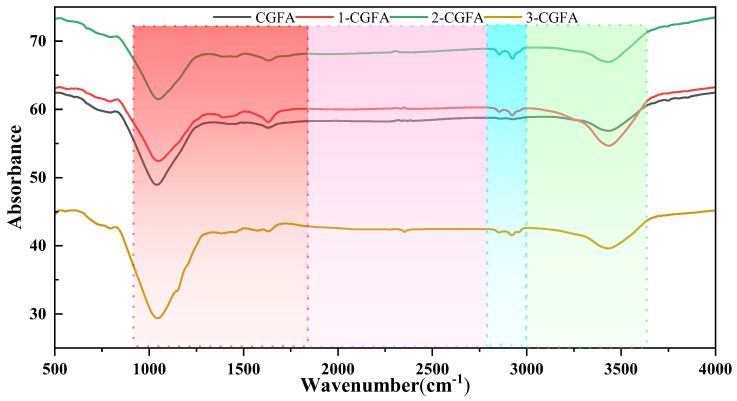
FTIR spectra of CGFA and alkylation modification.

As shown in Figure 4, the FTIR spectra of pristine CGFA and alkylated samples retain highly similar mineral framework features, indicating that alkylation mainly modifies the outer surface chemistry rather than substantially altering the inorganic matrix. In particular, a strong absorption band is consistently observed in the 900–1200 cm^−1^ region, which is commonly assigned to Si-O-Si and Si-O-Al stretching vibrations of silicate/aluminosilicate units. The comparable band position and profile across samples suggest that the mineral skeleton remains largely stable during alkylation. An absorption around 1600–1650 cm^−1^ is also present, and its intensity varies among samples, reflecting changes in oxygenated sites and the adsorbed-water state.

The most direct spectral evidence for alkyl chain introduction appears in the 2800–3000 cm^−1^ region. Compared with pristine CGFA, the modified samples show the emergence/enhancement of the characteristic aliphatic CH_2_/CH_3_ stretching doublet at 2920–2930 cm^−1^ and 2850–2860 cm^−1^, confirming successful incorporation and surface exposure of alkyl moieties. Table 2 shows that the C-H fraction increases from 24.84% to 43.91% after alkylation, supporting the assignment of the intensified C-H bands to surface alkyl groups. In addition, the broad band in the 3200–3600 cm^−1^ region is generally associated with O-H stretching of surface hydroxyls and adsorbed water. Its profile changes after alkylation, indicating an altered hydrogen-bonding/adsorbed-water environment due to surface polarity regulation. Consistently, XPS reveals a decrease in strongly polar carboxyl-type species from 7.93% to 1.58–5.47%, suggesting consumption transformation and shielding of polar oxygenated sites by hydrophobic chains, which is beneficial for lowering surface polarity and tuning wettability/dispersion behavior. Overall, FTIR captures the alkyl chain signature while maintaining stable mineral framework bands, providing spectroscopic evidence for surface alkylation-induced interfacial chemistry reconstruction. This directly proves that the alkyl chain has been successfully introduced. This corresponds to the increase in carbon and hydrogen content in ultimate analysis, indicating that the alkyl chain signal observed by infrared characterization is the main source of element increment.

#### 2.2.4. XRD Analysis

**Figure 5:**
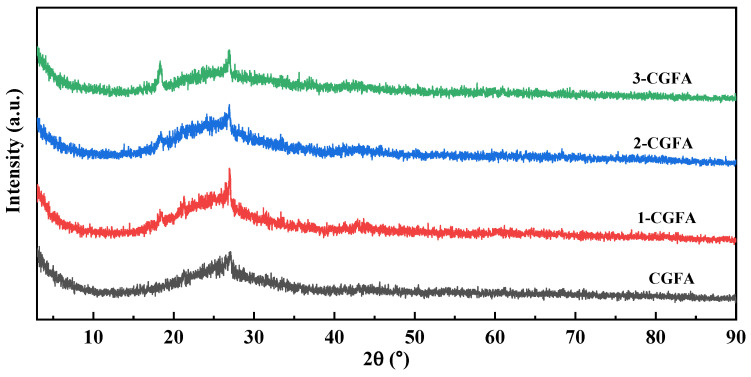
XRD spectra of CGFA and alkylation modification.

As shown in Figure 5, the XRD patterns of pristine CGFA and the alkylated samples over 2*θ* = 5–90° exhibit highly similar overall profiles, featuring a combination of a broad diffuse halo and several sharper crystalline peaks. This indicates the coexistence of substantial amorphous low-ordering components and a certain fraction of crystalline mineral phases. A pronounced broad halo is consistently observed in the 2*θ* = 15–35° range, which is typically associated with the scattering contribution from amorphous aluminosilicate glassy phases and disordered carbon structures commonly present in CGFA. Because the (002)-related scattering of disordered carbon often appears near 25–26° and may overlap with mineral reflections, the feature in this region is better interpreted as a superposition background from amorphous phases and low-ordering carbon, reflecting the intrinsic multiphase nature of CGFA. In addition to the diffuse halo, relatively sharp peaks are visible near 2*θ* = 20–21° and 26–27°, together with several weak reflections at higher angles. Such sharp peaks are commonly attributed to crystalline minerals frequently found in coal ash systems. Importantly, no new diffraction peaks appear after alkylation, and the main peak positions remain essentially unchanged across the series, suggesting that the alkylation treatment does not introduce detectable new crystalline phases nor induce apparent phase transformation of the mineral components. This is consistent with the fact that the introduced alkyl chains are expected to exist mainly as a low-loading amorphous organic layer on the surface, which typically does not generate distinct diffraction peaks.

The graphene-like carbon structure illustrated in Figure 1 represents the inherent aromatic carbon framework present in CGFA. During alkylation, grafting mainly occurs at edge sites and defect positions of these aromatic domains, forming C–O bonds and introducing alkyl chains while preserving the basic carbon skeleton. This structural feature supports the surface-selective modification mechanism and is consistent with the unchanged mineral phases observed by XRD.

Differences among samples are mainly reflected in relative peak intensities and background levels, especially in the low-angle region, implying that alkylation solvent processing may alter the relative surface exposure and scattering contribution of different components, while not changing the crystalline phase assemblage and the bulk framework. Differences among samples are mainly reflected in relative peak intensities and background levels, especially in the low-angle region, implying that alkylation solvent processing may alter the relative surface exposure and scattering contribution of different components, while not changing the overall amorphous character of the bulk matrix. Overall, the XRD patterns in Figure 5 demonstrate that the raw CGFA and all alkylated samples are predominantly amorphous, as evidenced by the broad diffuse halo in the 2θ = 15–35° range, with only a few weak and broad semi-crystalline reflections (20–21° and 26–27°) arising from minor crystalline mineral phases. Importantly, no new diffraction peaks appear after alkylation, and the overall amorphous character remains unchanged, indicating that the alkylation treatment does not introduce detectable crystalline phases nor alter the bulk amorphous mineral framework. Therefore, from a bulk structural perspective, alkylation primarily acts as an interfacial surface-level modification that preserves the intrinsic amorphous nature of the CGFA matrix, providing a structural basis for subsequent discussions combining surface-sensitive characterizations and interfacial behavior.

#### 2.2.5. Raman Analysis

**Figure 6:**
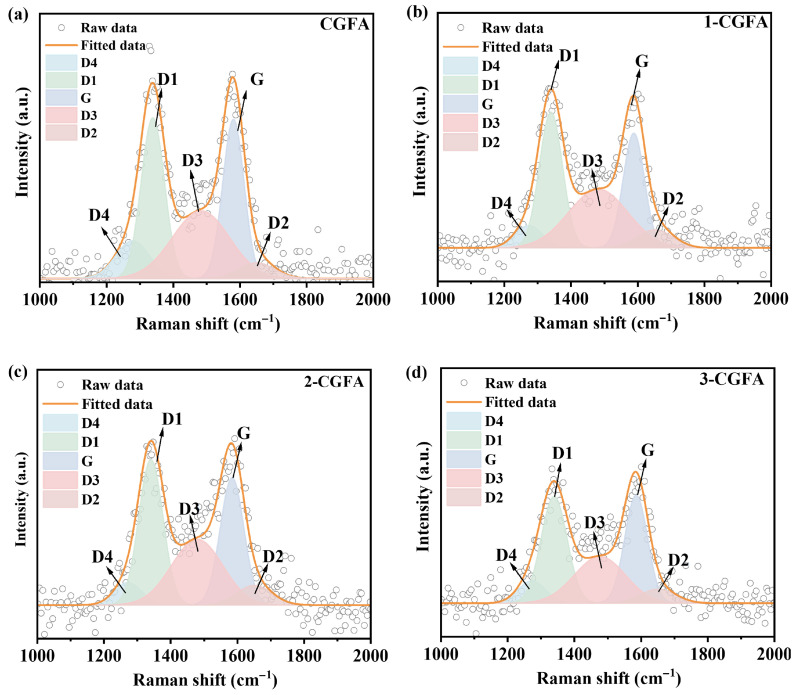
Raman spectrum of CGFA and alkylation modification: (**a**) CGFA, (**b**) 1-CGFA, (**c**) 2-CGFA, (**d**) 3-CGFA.

As shown in Figure 6, pristine CGFA and the alkylated samples exhibit typical Raman features of carbonaceous solids in the 1000–2000 cm^−1^ region. The spectra were deconvoluted into five bands centered at D4, D1, D3, G, and D2. The G band represents ordered sp^2^ carbon domains, whereas the D1 band is associated with disorder/defects [29,30,31,32,33]. The D2 band is commonly linked to defect edge-related contributions, the D3 band is typically assigned to amorphous or low-order carbon, and the D4 band is frequently used to describe contributions from more disordered/sp^3^- or heteroatom-affected carbon structures. The five-band fitting therefore indicates the coexistence of ordered sp^2^ domains and substantial disordered/defective carbon components in CGFA.

To minimize the influence of absolute intensity variations, peak-area ratios are emphasized. Table 3 summarizes the integrated areas and I_D_/I_G_, defined here as the area ratio D1/G. Pristine CGFA shows I_D_/I_G_ = 1.07. After alkylation, I_D_/I_G_ increases to 1.26 (1-CGFA), 1.14 (2-CGFA) and 1.1 (3-CGFA), corresponding to relative increases of 17.13%, 6.50%, and 2.21% versus CGFA. This trend suggests an overall increase in defect-related signatures at the Raman-probing scale, with 1-CGFA showing the most pronounced change, while 3-CGFA is closest to the pristine state.

Inspection of the fitted peak areas indicates that the increase in ID/IG is not solely caused by a stronger D1 band but also by a relative weakening of the G band. For example, in 1-CGFA, D1344 slightly decreases whereas G1581 decreases more substantially, leading to a marked rise in I_D_/I_G_. Moreover, the defect edge-related D2 band at 1652 cm^−1^ increases notably for 1-CGFA and 2-CGFA, and the corresponding D2/G ratio rises from 0.166 (CGFA) to 0.266 (1-CGFA) and 0.232 (2-CGFA), further supporting enhanced defect/edge contributions. The low-order carbon contribution reflected by D3 shows a non-monotonic behavior: 1-CGFA gives the highest D3/G (1.417), implying a larger relative fraction of amorphous/low-order carbon, whereas 3-CGFA shows a decreased D3/G, suggesting a reduced relative contribution of low-order carbon and/or an apparent attenuation due to surface-layer effects.

Overall, the Raman results demonstrate that alkylation modulates the defect characteristics and ordering of the carbonaceous phase in CGFA in a sample-dependent manner. The shift toward stronger defect-related features provides a microstructural basis for interpreting subsequent differences in thermo-oxidative behavior and kinetic parameters. The increased defect density indicated by the higher I_D_/I_G_ ratio aligns with the structural disruption caused by alkyl grafting, which is also reflected in the elevated carbon and hydrogen contents (Table 1). The consistent trends across structural and elemental analyses confirm the reliability of the alkylation modification.

#### 2.2.6. ^13^C NMR Analysis

**Figure 7:**
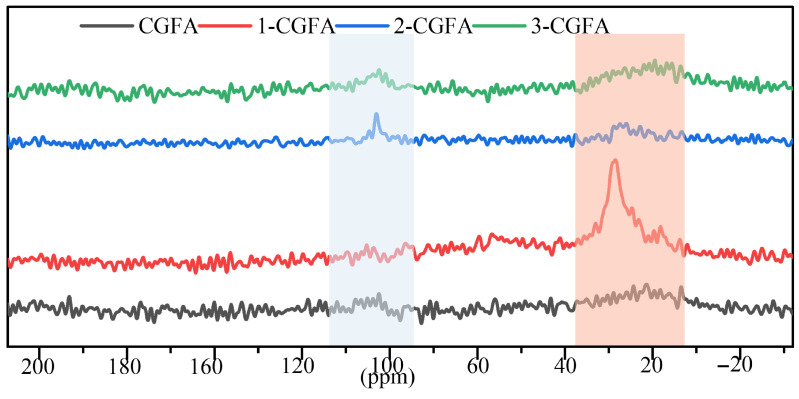
^13^C NMR spectrum of alkylated modified CGFA.

As shown in Figure 7, the solid-state ^13^C NMR spectra of the alkylated samples over 200 to −20 ppm display two major chemical-shift regions: a relatively concentrated signal in the 110–95 ppm range and prominent aliphatic carbon signals in the 35–10 ppm range. This spectral pattern indicates that the modified samples retain sp^2^-carbon environments associated with the original carbonaceous framework while exhibiting clearly detectable aliphatic carbons introduced by alkyl chains [34,35].

Sp^2^-carbon-related region: All samples show discernible features within the blue-shaded region, with the main intensity distributed around 100 ppm, suggesting the presence of sp^2^ carbon environments. The persistence of this region with only moderate variation across the series supports that alkylation mainly affects the outer interfacial/surface chemistry rather than reconstructing the bulk carbon framework.

Aliphatic alkyl carbon region: The most direct evidence of alkyl chain introduction appears in the orange-shaded region, which is typically assigned to aliphatic carbons such as CH_2_ and –CH_3_. Clear differences in relative intensity are observed among the samples: 2-CGFA exhibits the strongest and sharpest aliphatic signal centered at approximately 30 ppm, together with a pronounced enhancement in the 20–30 ppm range, indicating the highest apparent surface exposure visibility of alkyl chain carbons; 3-CGFA shows broader, more plateau-like enhancements across 35–10 ppm, implying overall enrichment of aliphatic carbons but with a more distributed chemical environment; 1-CGFA shows only weak variations in this region, suggesting the lowest aliphatic carbon contribution among the series.

Overall, the ^13^C NMR spectra provide direct spectroscopic evidence of alkyl chain incorporation via the distinct aliphatic carbon region, following an approximate trend of 2-CGFA > 3-CGFA > 1-CGFA, while the sp^2^-carbon-related signals (110–95 ppm) remain present in all samples. These observations are consistent with the notion that alkylation primarily modifies surface interfacial chemistry while largely preserving the sp^2^-carbon-dominated framework, and they can be cross-supported by the enhanced CH_2_/CH_3_ bands in FTIR and the increased C–H contribution in XPS. The distinct aliphatic carbon signals detected by ^13^C NMR correspond well to the increased carbon and hydrogen concentrations measured by elemental analysis (Table 2). This mutual verification demonstrates that alkyl chains have been effectively incorporated into CGFA.

### 2.3. Thermal Conversion Performance Analysis

#### 2.3.1. TG-DTG Analysis

**Figure 8:**
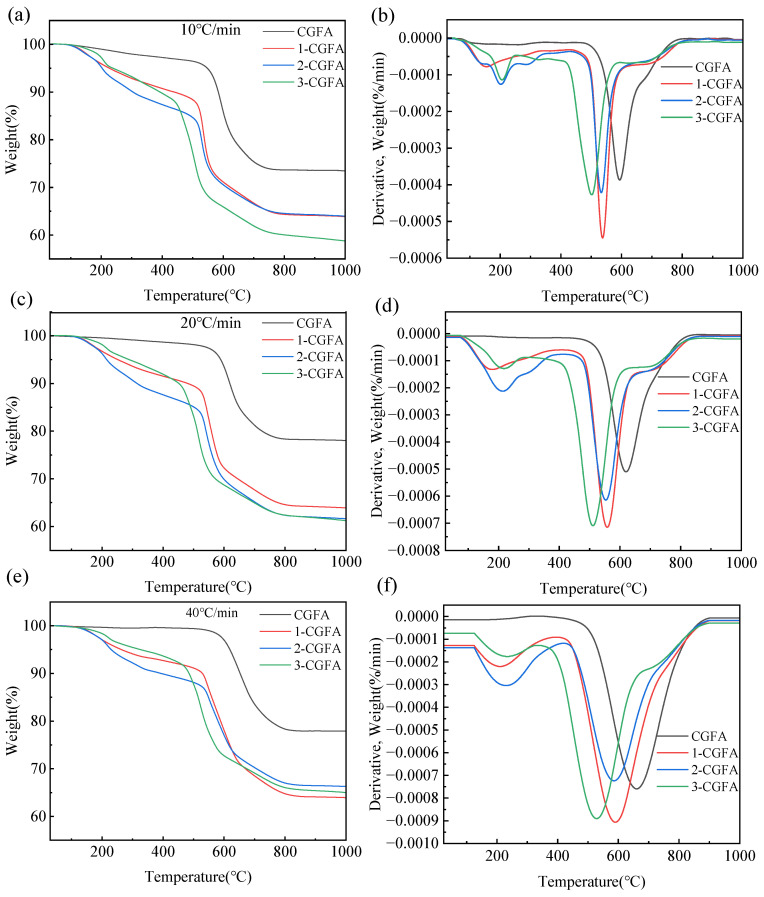
TG and DTG curve: (**a**) 10 °C/min TG, (**b**) 10 °C/min DTG, (**c**) 20 °C/min TG, (**d**) 20 °C/min DTG, (**e**) 40 °C/min TG, (**f**) 40 °C/min DTG.

Thermogravimetric analysis (Figure 8) clearly reveals the systematic influence of alkylation modification on the thermal oxidative behavior of CGFA. The raw CGFA exhibits its thermo-oxidative process under air atmosphere primarily in the high-temperature region, with an initial decomposition temperature of around 358 °C and a peak mass-loss rate at a relatively high temperature. In contrast, all alkylated samples 1-CGFA, 2-CGFA, and 3-CGFA display a distinct shift toward lower-temperature oxidation, with initial decomposition temperatures decreasing to 295–320 °C. The DTG peaks shift markedly to lower temperatures, accompanied by a notable increase in the maximum mass-loss rate. These observations indicate that alkylation effectively lowers the onset temperature of thermal oxidation, allowing the oxidative reaction to proceed under milder conditions while exhibiting a more concentrated and intense combustion process. Among the modified samples, 2-CGFA, functionalized with a long straight-chain alkyl group, shows the most pronounced low-temperature oxidation characteristics, highlighting the critical role of alkyl chain length and structure in tuning the thermal response behavior.

The enhanced thermo-oxidative reactivity of CGFA after alkylation can be fundamentally attributed to the reconstruction of its surface chemistry. The introduction of alkyl chains provides low-energy initiation sites for oxidation, where aliphatic structures can undergo cleavage and oxidation at relatively lower temperatures. The generated radicals or intermediates subsequently induce the oxidation of the neighboring aromatic carbon framework. The XPS results show that after alkylation, the C–H fraction increases significantly, while the content of strongly polar carboxyl groups (COO^−^) decreases from 7.93% to as low as 1.58%. The reduction in surface polarity facilitates oxygen adsorption and diffusion on the material surface. FTIR and ^13^C NMR further confirm the enrichment of aliphatic structures on the sample surface, with 2-CGFA exhibiting the strongest alkyl carbon signal. These chemical modifications shift the oxidation pathway from a high-temperature direct cleavage of aromatic structures in raw CGFA to a multistep reaction preferentially initiated by the grafted alkyl chains, thereby substantially reducing the apparent activation energy.

In addition to surface chemical modifications, alkylation also modulates the defect characteristics of the carbonaceous phase and the microscopic interfacial environment. Raman analysis reveals that the I_D_/I_G_ ratio generally increases after alkylation, with 1-CGFA exhibiting an increase of 17.13%, indicating an enrichment of defect sites that act as active centers to promote oxidation. Meanwhile, the steric occupation and surface coverage of alkyl chains alter the pore structure and gas diffusion pathways. Although the BET surface area decreases after modification, 2-CGFA maintains a relatively high pore volume of 0.40 cm^3^/g, which facilitates oxygen transport and volatile release.

#### 2.3.2. Kinetic Analysis

**Figure 9:**
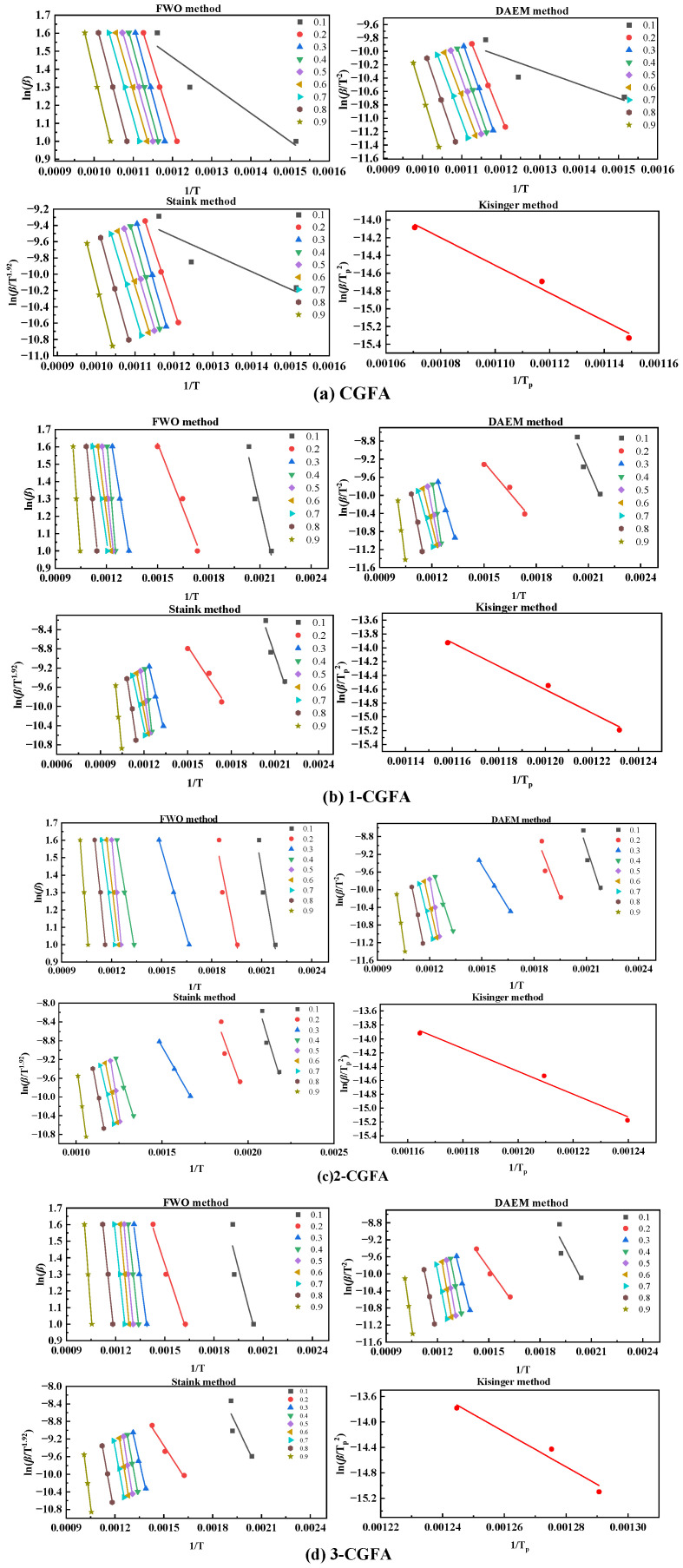
Estimated E values for four model-free methods for different values of *α*.

**Figure 10:**
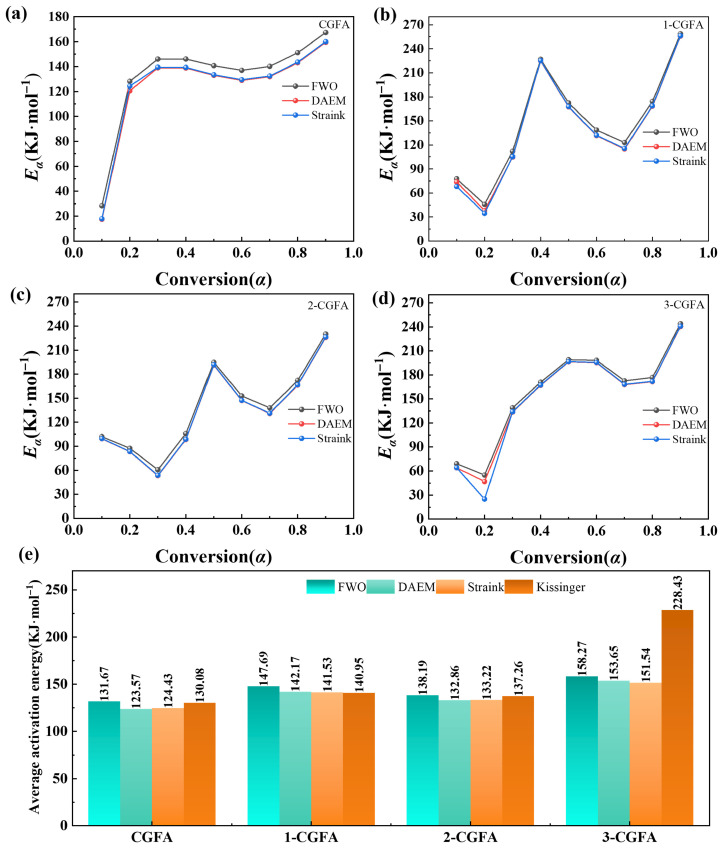
Four kinetic methods for calculating *E* for combustion in different samples: (**a**) CGFA (**b**) 1-CGFA (**c**) 2-CGFA (**d**) 3-CGFA (**e**) average activation energy of different models.

To quantitatively evaluate the effect of alkylation on the thermo-oxidative behavior of CGFA, the apparent activation energy *E* of each sample was calculated using model-free methods FWO, DAEM, Starink, and Kissinger. Although CGFA is a heterogeneous material containing both carbonaceous and mineral phases, the thermogravimetric experiments under oxidative atmosphere show a single dominant mass-loss stage (Figure 8), indicating that the overall oxidation process is kinetically controlled by a single rate-limiting step under the present conditions. Therefore, a simplified single-reaction model was adopted to obtain apparent kinetic parameters for comparative purposes across different alkyl chain lengths. This approach is widely accepted in the literature for evaluating the relative thermal reactivity of modified carbonaceous materials, as it provides consistent and interpretable trends without overfitting the data. The application of multiple model-free methods serves two purposes. First, it allows cross-validation of the calculated activation energies, as each method relies on different mathematical approximations: FWO and Starink use integral approaches with different temperature integral approximations, DAEM assumes a distribution of activation energies, and Kissinger is a peak-maximum method. Consistent results across these methods increase confidence in the kinetic parameters. Second, no single method is universally superior; rather, their suitability depends on the reaction characteristics. For CGFA which exhibits a single dominant mass-loss stage (Figure 9), all four methods yield comparable activation energy trends (Figure 9). Among them, the Starink method is generally considered the most accurate for solid-state reactions due to its better temperature integral approximation, while Kissinger is widely used for its simplicity but assumes a constant activation energy. Therefore, the average values derived from all four methods (Table 4) are reported to minimize method-induced bias. As shown in Figure 10, the evolution of *E* with conversion degree *α* varies significantly among the samples. The pristine CGFA exhibits relatively high and stable activation energy throughout the reaction, ranging from approximately 130 to 140 kJ/mol. The alkylated samples show a marked decrease in *E* at the initial stage, with 2-CGFA exhibiting an activation energy as low as approximately 95 kJ/mol, indicating that the grafted alkyl chains serve as low-energy initiation sites for oxidation. However, as the reaction proceeds to the middle and later stages, the activation energy of the alkylated samples gradually increases, resulting in higher average activation energy compared to pristine CGFA (Table 4). This phenomenon reflects the dual role of alkylation modification: the alkyl chains preferentially undergo oxidation, lowering the initiation barrier at the early stage; on the other hand, the surface coverage and steric hindrance introduced by the alkyl chains increase the diffusion resistance of oxygen at the carbon–mineral interface, leading to higher activation energy in the later stages. Furthermore, the effect of alkyl chain structure on activation energy exhibits a non-monotonic trend, 2-CGFA shows the lowest activation energy at the initial stage, while 3-CGFA exhibits a relatively smaller reduction, indicating that chain length and branching degree play important roles in regulating the reaction pathway.

At first glance, the higher average activation energy of alkylated samples (Table 4) appears to contradict their lower onset decomposition temperatures (Figure 8). However, this apparent discrepancy can be reconciled by examining the conversion-dependent evolution of E, as shown in Figure 10. For pristine CGFA, the activation energy remains relatively stable across the entire conversion range (approximately 130–140 kJ/mol), indicating that a single rate-limiting step dominates the whole oxidation process. In contrast, alkylated samples exhibit a two-stage kinetic behavior:

Low-conversion stage (*α* < 0.3): The grafted alkyl chains serve as low-energy initiation sites that undergo preferential oxidation. This is evidenced by the markedly lower *E* values in this initial stage, which directly explains the earlier onset of mass loss observed in Figure 8.

High-conversion stage (*α* > 0.4): After the consumption of alkyl chains, further oxidation of the aromatic carbon framework becomes rate-limiting. Meanwhile, the surface coverage and steric hindrance introduced by alkylation increase the diffusion resistance of oxygen at the carbon–mineral interface. Consequently, *E* rises significantly in this later stage, pulling up the overall average activation energy.

Therefore, the higher average *E* for alkylated samples does not indicate intrinsically lower reactivity. Instead, it reflects a shift from a single-step, high-temperature pathway to a multistep pathway involving low-energy initiation followed by diffusion-limited oxidation. This mechanistic interpretation resolves the apparent contradiction and highlights the importance of conversion-dependent kinetics for heterogeneous materials like CGFA.

Despite the comparable alkyl grafting levels between 2-CGFA and 3-CGFA, the branched-chain-modified 3-CGFA exhibits consistently higher activation energy than the straight-chain-modified 2-CGFA (Table 4). This counterintuitive phenomenon can be primarily attributed to two factors. First, steric hindrance from the branched alkyl chains in 3-CGFA creates a disordered and bulky surface layer that impedes oxygen accessibility to the reactive C-H sites, diminishing their effectiveness as low-energy oxidation initiation points. Second, pore structure analysis (Figure 1) reveals that 3-CGFA suffers from more severe pore blockage compared to 2-CGFA, which restricts oxygen diffusion into the intraparticle network and elevates mass-transfer resistance during oxidation.

Furthermore, Raman spectroscopic evidence (Table 3) shows that 3-CGFA exhibits the smallest increase in I_D_/I_G_ ratio (only 2.21%) among all modified samples, whereas 2-CGFA achieves a 6.50% increase. This indicates that branched alkylation is less effective in generating defect sites on the carbonaceous framework defects that typically serve as additional oxidation hotspots. Collectively, the combination of steric hindrance, pore blockage, and limited defect enrichment explains why 3-CGFA, despite substantial aliphatic grafting, fails to achieve the same low-temperature reactivity enhancement as 2-CGFA, highlighting that alkyl chain configuration is as critical as total grafting density in governing thermal response.

The Raman I_D_/I_G_ ratio is closely correlated with the kinetic parameters of thermal oxidation. The 17.13% increase in I_D_/I_G_ for 1-CGFA indicates significantly enhanced carbon defect sites, which serve as active centers for oxidation initiation. Consequently, 1-CGFA exhibits a lower activation energy *E* at low conversion, consistent with the promoted low-temperature reactivity induced by abundant structural defects. This structure–kinetic relationship confirms that higher defect density effectively reduces the initial energy barrier and accelerates thermo-oxidative reactivity.

#### 2.3.3. Thermodynamic Analysis

**Figure 11:**
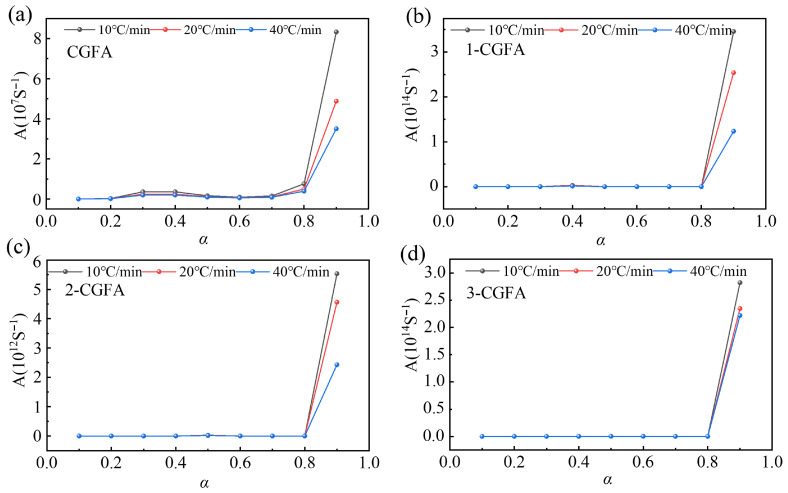
Finger front factors *A* different for samples with different heating rates: (**a**) CGFA, (**b**) 1-CGFA, (**c**) 2-CGFA, (**d**) 3-CGFA.

The pre-exponential factor *A* is a critical kinetic parameter that reflects the collision frequency and mechanistic complexity of the reaction system, and its variation can reveal the impact of alkylation on the reaction mechanism. As shown in Figure 11, the pristine CGFA exhibits *A* values at a certain level across different heating rates. After alkylation, the *A* values of all modified samples increase significantly, with 2-CGFA showing the most pronounced increase. This increasing trend indicates that the molecular collision frequency of the reaction system is significantly enhanced after alkylation, leading to improved reaction activity.

The remarkable nine-order-of-magnitude increase in the pre-exponential factor A for alkylated samples should be interpreted primarily as a kinetic compensation effect rather than a genuine change in intrinsic molecular collision frequency. For heterogeneous materials like CGFA, the apparent kinetic parameters *A* and *E* commonly exhibit a linear compensation relationship: higher apparent activation energy is accompanied by a higher pre-exponential factor. This phenomenon arises because alkylation modifies the surface structure, active site distribution, mass transfer pathways, and interfacial environment, rather than altering the fundamental collision frequency of gas solid reactions. The large increase in A reflects the expanded number of available reaction sites, altered surface adsorption–desorption behavior, and modified diffusion resistance introduced by grafted alkyl chains, rather than a true increase in collision frequency. This kinetic compensation behavior is widely reported in heterogeneous thermal oxidation systems, consistent with the complex multiphase nature of CGFA.

The introduction of alkyl chains forms an organic coverage layer on the CGFA surface, altering the contact mode between reactant molecules (such as oxygen) and active sites, thereby increasing the probability of effective collisions. Second, the alkyl chains act as preferential oxidation sites, shifting the reaction pathway from single-step high-temperature carbon oxidation to a multistep process. Although this increases mechanistic complexity, it also provides more reaction channels and collision opportunities for the reaction. Third, the reduced surface polarity may promote oxygen adsorption and diffusion on the material surface, further enhancing molecular collision efficiency.

Although the average activation energy *E* of the alkylated samples increases to some extent, the pre-exponential factor A also increases significantly. According to the Arrhenius equation, the reaction rate constant k is influenced by both A and *E*. While the increase in activation energy has an inhibitory effect on the reaction rate, the significant increase in the pre-exponential factor plays a compensatory role, allowing the overall reaction rate to be maintained or even enhanced. Consequently, in the thermogravimetric experiments, the alkylated samples exhibit lower onset decomposition temperatures (decreasing from 358 °C to 295 °C) and higher maximum mass-loss rates, indicating significantly enhanced combustion performance. This synergistic regulation mechanism reveals that alkylation modification achieves precise control over the thermo-oxidative behavior of CGFA by simultaneously modulating both the activation energy and the pre-exponential factor: the alkyl chains act as the initiation barrier by altering the reaction pathway, while also enhancing the overall reaction activity by increasing molecular collision frequency.

This compensation effect is not unique to our system. Hayashi et al. [25] reported analogous behavior in alkylated low-rank coals, where longer alkyl chains simultaneously enhanced volatile yields (implying altered apparent reactivity) while increasing the complexity of the reaction network. Similarly, Yang et al. [27] observed concurrent increases in E and A after O-alkylation of coal, attributing this to the kinetic compensation effect arising from surface reconstruction and modified active site accessibility. Thus, the nine-order-of-magnitude increase in A for 2-CGFA, accompanied by a moderate increase in average E, reflects a genuine compensation mechanism rather than an experimental artifact, and is characteristic of heterogeneously confined oxidation on alkyl-grafted surfaces.

### 2.4. Summary of Structure–Property Relationship and Application Prospects

In summary, alkylation modification, through precise molecular design, grafts aliphatic side chains onto the stable aromatic backbone of CGFA, achieving a transition from thermally stable to controllably thermally responsive. The core structure–property relationship is described as follows: alkyl chains act as molecular fuses and structural loosening agents. By lowering the initial reaction energy barrier and altering the internal mass transfer environment, they enable predictable and tunable down-regulation of the thermal oxidation behavior. Based on this clear structure–property relationship, this work opens up the following promising directions for the high-value utilization of CGFA.

(1) Controlled Pyrolysis for Functional Carbon Materials.

By adjusting the length and grafting density of alkyl chains, the decomposition temperature range and volatile release rate of the precursor during pyrolysis can be precisely controlled. This is crucial for fabricating carbon materials with specific pore structures, high specific surface area, or tailored surface chemistry, optimizing their performance in applications such as supercapacitors, adsorption, or catalysis.

(2) Flame-Retardant Synergist or Char-Forming Agent in Polymer Composites.

The lower thermal stability and controlled decomposition behavior make alkylated CGFA a potential novel intelligent char-forming agent. In the early stages of a fire, it can decompose earlier, releasing inert gases and promoting the formation of a dense, continuous protective char layer, thereby enhancing the flame retardancy of polymer composites. Its decomposition kinetics can be designed to match the thermal decomposition of the matrix resin, achieving optimal synergistic flame-retardant effects.

(3) Precursor for Energy Materials.

The controllable oxidation behavior makes it suitable as a host material for lithium–sulfur batteries or a hard carbon precursor for sodium-ion batteries. The early formation of abundant pores and active surfaces can facilitate sulfur loading and anchoring of polysulfides, or be beneficial for sodium ion intercalation and adsorption.

(4) Construction of Responsive Materials.

Utilizing its thermal responsiveness, alkylated CGFA could serve as a thermally triggered release carrier or a sensing element in temperature-sensitive sensors, undergoing controlled structural disintegration or property changes at specific temperature thresholds.

## 3. Sample and Experimental Methods

### 3.1. Alkylation Experiment

**Figure 12:**
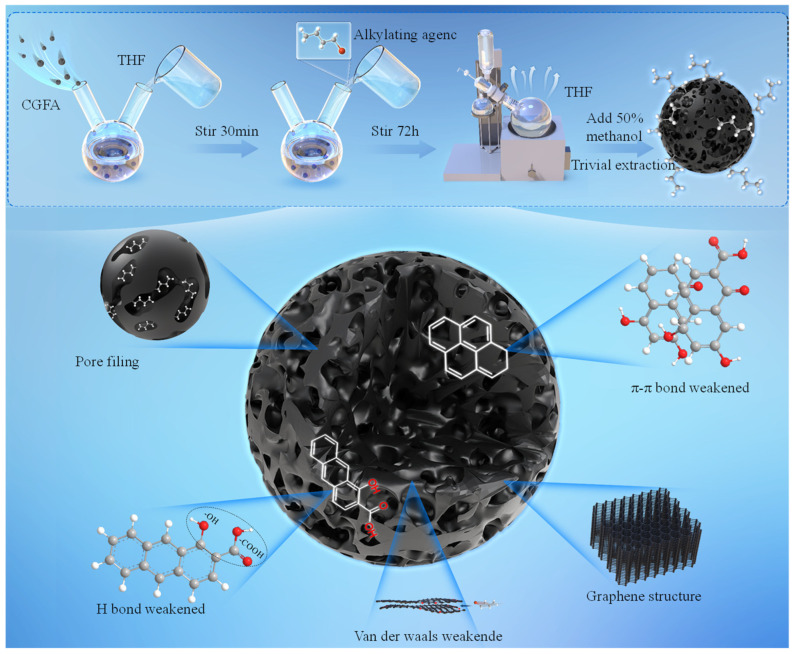
Alkylation experimental process and mechanism.

The raw CGFA was collected from an industrial entrained-flow coal gasification plant. The parent coal was fed into the gasifier and reacted with oxygen and steam at high temperature and high pressure to produce syngas, during which CGFA was generated as a fine solid waste byproduct. A total of 5 g of CGFA sample was added to two flasks, and 150 mL of tetrahydrofuran solution was poured into the flasks. Argon gas was continuously introduced into the flasks at a rate of 500 mL/min and stirred magnetically at room temperature for 30 min. Then, 14.6 mL of tetrabutylammonium hydroxide aqueous solution (1.54 mol/L) was slowly added into the flask and stirred for 2 h under argon protection. Afterwards, 22.5 mmol of alkylating agent was added dropwise to the flask and the mixed solution was stirred for 72 h. Then, dilute hydrochloric acid was slowly added dropwise until the solution became neutral, and a rotary evaporator was used to remove THF at 60 °C; using a 50% methanol aqueous solution as the extraction solvent, the sample was extracted using a soxhlet extractor for a period of 6 days. Silver nitrate titration was used to extract the liquid and check for the presence of halide ions. Sodium tetraphenylborate was used to detect the presence of tetrabutylammonium ions. The specific research process is shown in Figure 12. The sample extracted by soxhlet extraction is vacuum dried at 100 °C for 2 days [28,36,37,38,39]. 1-bromobutane, 1-bromohexane, 1-bromo-4-methylpentane, and tetrahydrofuran were all purchased from Aladdin Reagent (Shanghai) Co., Ltd. The basic information for different alkylating reagents is shown in Table 5.

Adding alkaline reagents can neutralize acidic groups in CGFA, causing these groups to lose H^+^ and generate negatively charged CGFA polyanions, increasing its active sites. The alkyl groups in the molecule will undergo substitution reactions with negatively charged CGFA polyanions, attaching the alkyl groups to CGFA molecules to complete the modification.

### 3.2. Ultimate Analyses Experiments

In this study, we employed the EA3000 automatic elemental analyzer to determine the C, H, N, and S contents of both raw CGFA and its alkylated derivatives. Prior to measurements, we calibrated the instrument using certified reference materials to ensure an analytical uncertainty ≤0.5%. By analyzing the elemental compositional changes, we investigated the impact of alkylation on the organic moieties of CGFA, thereby providing a quantitative foundation for its physicochemical characterization.

### 3.3. Characterization Experiments

This study characterized the physicochemical structures of raw CGFA and CGFA modified with different alkylating reagents, analyzing the structural changes in the materials before and after alkylation modification through the following testing methods.

Nitrogen adsorption–desorption measurements were performed at 77 K using a Quadrasorb EVO surface area and pore size analyzer (Quantachrome, Boynton Beach, FL, USA). Samples were degassed at 120 °C for 6 h prior to testing to remove adsorbed moisture and impurities. Specific surface area was calculated via the BET model, while pore size distribution and pore volume were derived from the Barrett–Joyner–Halenda (BJH) model based on the desorption branch.

FTIR spectra were recorded on a TENSOR II (Bruker, Karlsruhe, Germany) in the range of 4000–400 cm^−1^. Samples were mixed with KBr and pressed into pellets, with a resolution of 4 cm^−1^ and 32 scans accumulated for each spectrum to improve signal-to-noise ratio.

XPS was carried out on an ESCALAB 250Xi spectrometer (Thermo Fisher Scientific, Waltham, MA, USA) using Al Kα radiation as the excitation source. The pass energy was set to 20 eV for high-resolution scans and 100 eV for survey scans. Binding energy was calibrated with the C1s peak at 284.8 eV as the reference. Peak fitting was performed using XPS Peak 4.1 software with a Shirley-type background correction.

X-ray diffraction patterns were collected on a Bruker D8 Advance diffractometer (Bruker, Karlsruhe, Germany) with Cu Kα radiation. The operating voltage and current were 40 kV and 40 mA, respectively. Data were recorded in the 2θ range of 5–90° with a step size of 0.02° and a scanning speed of 5°/min.

Raman spectra were obtained using a Horiba LabRAM HR Evolution spectrometer (HORIBA, Villeneuve-d Ascq, France) with a 532 nm laser excitation source. The laser power was set to 5 mW to avoid sample decomposition, and spectra were recorded in the range of 1000–2000 cm^−1^ with a spectral resolution of 2 cm^−1^.

^13^C NMR spectra were acquired on a Bruker AVANCE III 400 MHz spectrometer (Bruker, Germany). The spinning speed was 10 kHz, contact time was 2 ms, and relaxation delay was 2 s. A total of 1024 scans were accumulated for each sample to ensure sufficient signal intensity.

### 3.4. Thermogravimetric Experiment

This study obtained TG and DTG curves of different samples through enthusiastic experimentation. Thermogravimetric experiments were conducted using Thormogravimetric Analysts TGA/DSC1 from Mettler Toledo, Greifensee, Switzerland. For each experiment, 10 mg of the sample was placed in the analyzer. The initial temperature was set to 25 °C, the heating rates were 10, 20 and 30 °C/min respectively, and the final temperature was 1000 °C. The experimental atmosphere was air, and the gas flow rate was 40 mL/min.

### 3.5. The Kinetics Calculation

#### Kinetic Model and Parameter Definitions

The thermal oxidative decomposition of carbonaceous materials can be described by the general solid-state reaction kinetic equation [40,41], which correlates the reaction rate with temperature and conversion degree:

(1) $$dt/d\alpha =k\left(T\right)\cdot f\left(\alpha \right)$$

(2) $$f\left(a\right)=1-\alpha^{\prime \prime }$$

where *α* represents the fraction of the sample that has undergone thermal decomposition at time t, defined as

(3) $$\alpha =\frac{m_{0}-m_{t}}{m_{0}-m_{f}}$$

In this equation, *m*_0_ is the initial mass of the sample, *m*_t_ is the mass of the sample at time t, and *m_f_* is the final residual mass of the sample. The value of *α* ranges from 0 to 1.

*k*(*T*) is the temperature-dependent rate constant, which follows the Arrhenius equation:

(4) $$k\left(T\right)=A\cdot \exp\left(-\frac{E}{RT}\right)$$

*A* is the pre-exponential factor, *E* is the apparent activation energy (kJ/mol), *R* is the universal gas constant (8.314 J/(mol·K)), and *T* is the absolute temperature (K).

*f* (*α*) is the reaction model function, which describes the relationship between the reaction rate and conversion degree. Its form depends on the rate-limiting step of the thermal decomposition reaction.

*A* can be obtained according to the model-free method calculation in Formula (5) for the estimation of *E*_α_; T_p_ is taken as the temperature corresponding to the peak of the DTG curve:

(5) $$A=\left(\beta \cdot E\cdot \exp\left(\frac{E}{RT_{p}}\right)\right)/\left(RT_{p}^{2}\right)$$

By substituting the Arrhenius equation into Equation (1) and considering the linear heating rate $\beta =\frac{dt}{dT}$, Equation (1) can be rearranged into an integral form by separating variables and integrating with respect to temperature:

(6) $$\int_{0}^{\alpha }\frac{d\alpha }{f\left(\alpha \right)}=\int_{T_{0}}^{T}\frac{A}{\beta }\times \exp(-\frac{E}{RT})dT$$

where $G\left(\alpha \right)=\int_{0}^{\alpha }\frac{d\alpha }{f\left(\alpha \right)}$ is the integral form of the reaction model function f(α), and *T*_0_ is the initial temperature.

The kinetic calculation of coal gasification fine ash was carried out by FWO, DAEM, Starink and Kissinger methods. The four model-free methods mentioned are Equations (7)–(10), and the *E* is given by their slopes of the straight line in Equations (7)–(10) [42]:

FWO:

(7) $$\mathrm{lg}\beta =\mathrm{lg}\left(\frac{AE}{Rg\left(\alpha \right)}\right)-2.315-0.4567\frac{E}{RT}$$

DAEM:

(8) $$\ln\left(\frac{\beta }{T^{2}}\right)=\ln\left(\frac{AR}{E}\right)+0.6575-\frac{E}{RT}$$

Starink:

(9) $$\ln\left(\frac{\beta }{T^{1.92}}\right)=C_{\delta }-1.0008\frac{E}{RT}$$

Kissinger:

(10) $$\ln\left(\frac{\beta }{T_{p}^{2}}\right)=\ln\left(\frac{AR}{E}\right)-\frac{E}{RT_{p}}$$

## 4. Conclusions

This study systematically investigates the regulation mechanism of alkyl chain length on the physicochemical structure and thermo-oxidative behavior of coal gasification fine ash through surface alkylation with reagents of varying chain lengths. The main conclusions are as follows:

(1) Alkylation successfully achieves chemical grafting of aliphatic chains onto the CGFA surface. The hydrogen content increases by 80–164%, the surface C–H fraction rises from 24.84% to 43.91%, and polar carboxyl groups decrease from 7.93% to 1.58%, indicating a significant reduction in surface polarity.

(2) Alkylation significantly affects carbon defect structures and pore characteristics. The I_D_/I_G_ ratio increases by up to 17.13% after modification, while long straight-chain alkyl groups maintain a relatively high pore volume of 0.40 cm^3^/g. In contrast, short-chain and branched-chain modifications reduce the specific surface area to 280–380 m^2^/g.

(3) Alkylation substantially enhances the combustion performance of CGFA. The grafted alkyl chains serve as molecular fuses that ignite at lower temperatures, promoting earlier and more intense oxidation. The onset decomposition temperature decreases from 358 °C to 295–320 °C, and the maximum mass-loss rate increases markedly, indicating a more concentrated and efficient combustion process.

(4) Long straight-chain alkyl groups demonstrate optimal performance in lowering the onset oxidation temperature, maintaining pore structure, and enhancing reaction activity, whereas branched-chain structures exhibit a diminished regulatory effect due to steric hindrance, revealing the synergistic mechanism of chain length and configuration on the interfacial reaction pathway.

## Figures and Tables

**Table 1:** Ultimate analyses of sample.

Sample	Ultimate Analyses (wt%)			
	C	H	N	S
CGFA	22.03	0.50	0.10	0.09
1-CGFA	24.48	0.90	0.11	0.11
2-CGFA	28.97	1.32	0.18	0.12
3-CGFA	24.02	1.08	0.16	0.11

**Table 2:** Relative content of carbon functional groups in different samples.

Sample	Relative Content of Surface Functional Groups			
	C-C	C-H	C-O	COO^−^
CGFA	65.53	24.84	1.7	7.93
1-CGFA	47.22	30.33	18.96	3.49
2-CGFA	45.05	43.91	9.47	1.58
3-CGFA	40.24	37.69	18.59	5.47

**Table 3:** Raman integration area.

	D4	D1	D3	G	D2	ID/IG
CGFA	1231.6	3623.8	3831.3	3376.9	560.9	1.07
1-CGFA	676.7	3446.2	3884.3	2741.7	729.2	1.26
2-CGFA	676.7	3870.8	3737.1	3386.9	785.3	1.14
3-CGFA	676.7	3052.9	2653.8	2783.5	560.9	1.1

**Table 4:** Average activation energy of different samples.

Sample	CGFA	1-CGFA	2-CGFA	3-CGFA
Average *E* (kJ/mol)	131.67	147.69	138.19	158.27

**Table 5:** Basic information for different alkylating reagents.

Reagent Name	Structural Formula	Alkylated Sample Abbreviation
1-bromobutane	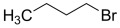	1-CGFA
1-bromoheptane	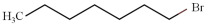	2-CGFA
1-Bromo-4-methylpentane	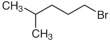	3-CGFA

## Data Availability

The original contributions presented in this study are included in the article/Supplementary Materials. Further inquiries can be directed to the corresponding author(s).

## Supplementary Materials

The following supporting information can be downloaded at: https://www.mdpi.com/article/10.3390/molecules31101682/s1, Figure S1: BET curve; Figure S2 Changes in specific surface area and pore volume and after CGFA modification; Figure S3 SEM images; Figure S4 XPS Survery.
