# Supplementary material for: Tuning Thermochemistry Behavior of Coal Gasification Fine Ash via Alkyl Chain-Length-Dependent Surface Functionalization: Mechanisms and Structure–Property Relationships
Source: Molecules. 2026 May 15;31(10):1682. doi: 10.3390/molecules31101682 (PMC13209994; doi:10.3390/molecules31101682)
Supplement: Supplementary file 1 [file molecules-31-01682-s001.zip › molecules-4248691-supplementary.pdf]

Supplementary File(s)

# Tuning thermochemistry behavior of coal gasification fine ash via alkyl chain length dependent surface functionalization: mechanisms and structure property relationships

Luzhen Jiao[1], Huiguo Yu[1], Yanshun Li[1], Yiqun Chen[1], Jiawei Li[*2], Xiaoguang Li[*3]

*1 Shandong Vocational College of Science and Technology*

*6388, West Ring Road, WeiFang 261053, P.R. China*

*2 School of Energy and Power Engineering, Northeast Electric Power University*

*169, Chang Chun Street, JiLin132012, P.R. China*

*3 School of Energy and Environmental Engineering, University of Science and Technology Beijing*

*30, Xueyuan road, Beijing 100083, P.R. China*

**Corresponding author:* Jiawei Li[*2]

*E-mail: ljweducation@126.com*(J. Li).

**Alkylation reaction**

Alkylation reaction refers to the process of transferring and connecting alkyl groups (such as methyl $CH_3$, ethyl $C_2H_5$) in alkylating agents to carbon, oxygen, nitrogen and other atoms in target organic compounds through chemical reactions under specific conditions such as catalysts. Its essence is "alkyl transfer and grafting", with the core purpose of changing the structure of the target molecule, adjusting its physical and chemical properties such as solubility, stability, and reactivity to meet different application needs. According to the different atoms connected to the alkyl group, it is mainly divided into three types: C-alkylation, O-alkylation, and N-alkylation. Most reactions require acid or base catalysts, and environmental parameters such as temperature, pressure, and solvent are controlled to ensure efficiency and selectivity.

Selective O-alkylation modification is accompanied by a significant amount of electron transfer, free radical generation, ether bond cleavage, and weak C-C bond cleavage. This process will lead to changes in the configuration of coal molecules and further induce changes in the migration pathway of fuel nitrogen during coal thermal conversion. In addition, the abundant free radicals during selective O-alkylation will attack the aromatic layer, leading to the generation of a large number of dangling bonds, and also causing the aromatic layer to tend towards miniaturization and curling, which is beneficial for the improvement of the lattice activity of coal coke in the later stage[41,42].

**Alkylation modified coal gasification fine ash experiment**

This article conducts grafting experiments on coal gasification fine ash using four different alkyl reagents. The hydrogen atoms on acidic sites (hydroxyl groups and carboxyl groups) are replaced by basic ions to form coal polyanions. Subsequently, the coal polyanions are attacked by the carbon atoms on the alkyl halides, completing the alkylation substitution.

The specific experimental process is as follows:

5g of CGFA samples were added to each of the two-necked flasks, and 150mL of tetrahydrofuran solution were poured into the flasks. Argon gas was continuously introduced into the flasks at a rate of 500mL/min, while magnetic stirring was

performed at room temperature for 30 minutes. 14.6 ml of tetrabutylammonium hydroxide aqueous solution (with a concentration of 1.54mol/L) were slowly added to the flasks, and continue stirring for 2 hours under argon protection. Afterwards, 22.5 mmol of alkylating reagent was added dropwise to the flasks, and stirring of the mixed solution were continued for 72 hours. Subsequently, dilute hydrochloric acid is slowly added dropwise until the solution becomes neutral, and tetrahydrofuran (THF) was removed using a rotary evaporator at 60°C. With a 50% methanol aqueous solution as the extraction solvent, the sample was extracted using a Soxhlet extractor for 6 days. The extract was detected using the silver nitrate titration method to confirm the presence of halide ions; meanwhile, sodium tetraphenylborate was used to detect the presence of tetrabutylammonium ions. The sample after Soxhlet extraction was dried under vacuum at 100°C for 2 days. The specific reaction chemical formula is as follows:

$$\text{CGFA-OH+B} \rightarrow \text{CGFA-O}^{-}\text{+BH}^{+} \quad (1)$$

$$\text{CGFA-COOH+B} \rightarrow \text{CGFA-COO}^{-}\text{+BH}^{+} \quad (2)$$

$$\text{CGFA-O}^{-}\text{+RX} \rightarrow \text{CGFA-OR+X}^{-} \quad (3)$$

(1) Argon gas is an inert gas, continuously introduced at a rate of 500mL/min, which can completely drive away the air in the flask, prevent the active groups in CGFA from being oxidized, or prevent side reactions between water vapor and reagents.
(2) Tetrabutylammonium hydroxide aqueous solution is an alkaline reagent whose $OH^-$ can neutralize acidic groups (such as-OH,-COOH) in CGFA, causing these groups to lose $H^+$ and generate negatively charged “CGFA polyanions”; Stirring under argon protection for 2 hours can ensure that the alkaline reagent fully reacts with CGFA, allowing more active sites to be activated while avoiding the destruction of the generated anion structure by components in the air.
(3) Alkylation reagents are alkyl donors, and the alkyl groups in their molecules will

undergo substitution reactions with negatively charged CGFA polyanions, attaching the alkyl groups to CGFA molecules to complete modification. Dropwise addition of reagents can avoid side reactions caused by high local concentrations. Long term stirring for 72 hours can make the reaction more complete, improve alkylation efficiency and modification uniformity.

(4) Neutralization with dilute hydrochloric acid: neutralize unreacted tetrabutyl hydroperoxide while terminating residual alkaline catalytic reactions; 60 ℃ rotary evaporation to remove THF: THF has a boiling point of about 66 ℃, and at 60 ℃, it is evaporated under reduced pressure to dissolve, leaving the alkylated CGFA in a solid state.

(5) 50% methanol aqueous solution is the extraction solvent: it can dissolve unreacted alkylating reagents, remaining tetrabutylammonium hydroxide and its by-products, but does not dissolve large molecule alkylated CGFA.

(6) Silver nitrate titration for measuring halide ions: Alkylation reagents often contain halogen atoms. If there are no halide ions in the extraction solution, it indicates that the unreacted alkylating reagents have been removed completely.

(7) Sodium tetraphenylborate detection of tetrabutylammonium ion: If no positive result is detected, it indicates that excess tetrabutylammonium hydroxide has been removed to avoid impurities affecting product performance.

**Sample introduction**

(1) CGFA is original sample of coal gasification fine ash

(2) 1-CGFA is coal gasification fine ash modified with 1-bromobutane.

(3) 2-CGFA is coal gasification fine ash modified with 1- bromoheptane.

(4) 3-CGFA is coal gasification fine ash modified with 1-Bromo-4-methylpentane.

## Table of Contents

**Figure S1** BET curve

**Figure S2** Changes in specific surface area and pore volume and after CGFA modification

**Figure S3** SEM images

**Figure S4** XPS Survery

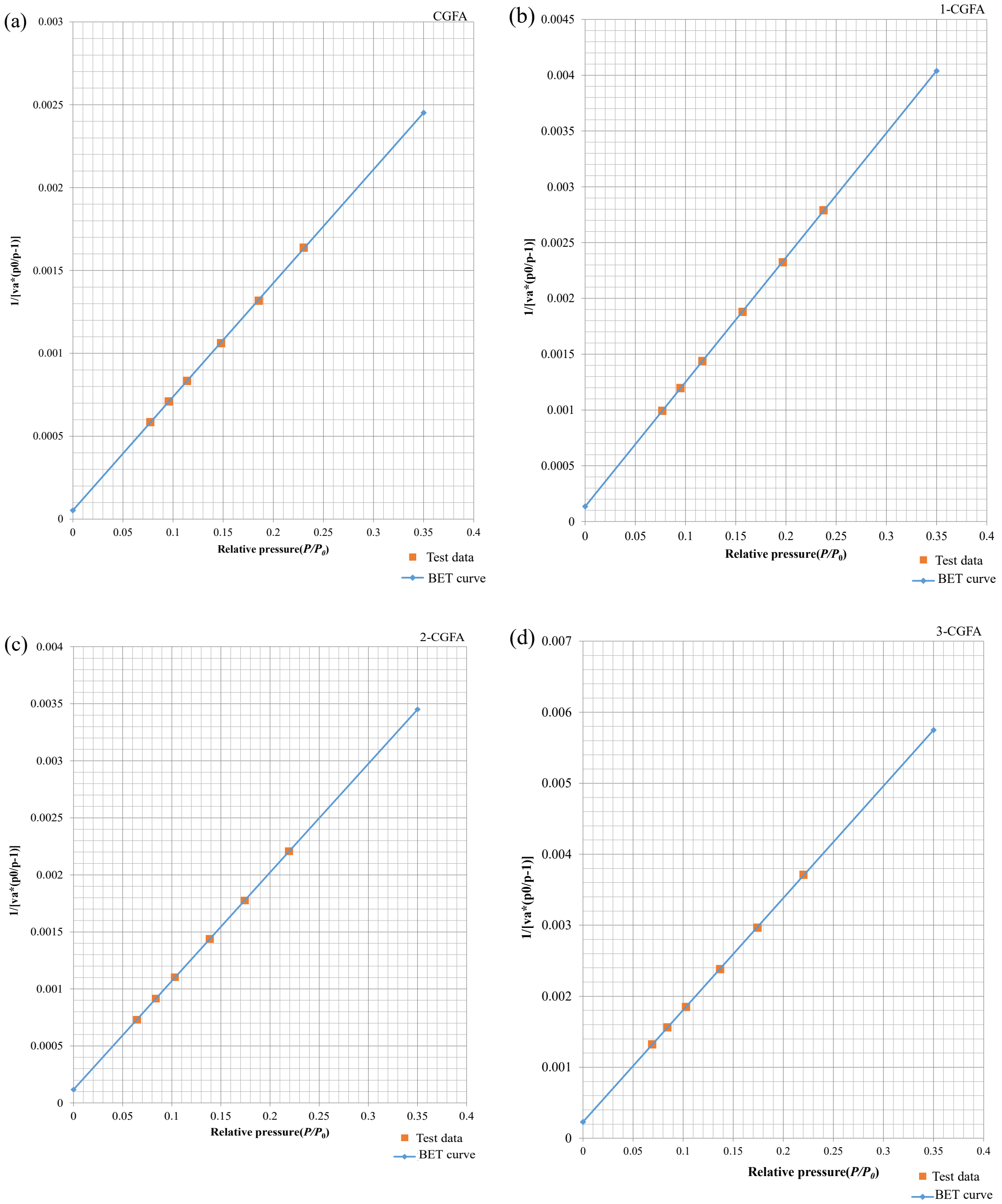

(a)
CGFA
1/[va*(p0/p-1)]
Relative pressure(P/P0)
Test data
BET curve
(b)
1-CGFA
1/[va*(p0/p-1)]
Relative pressure(P/P0)
Test data
BET curve
(c)
2-CGFA
1/[va*(p0/p-1)]
Relative pressure(P/P0)
Test data
BET curve
(d)
3-CGFA
1/[va*(p0/p-1)]
Relative pressure(P/P0)
Test data
BET curve

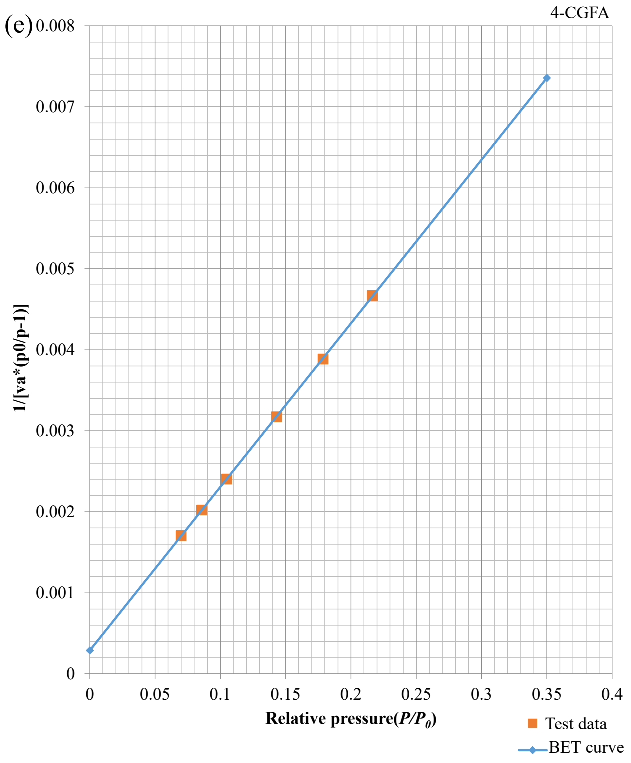


**Figure S1** BET curve

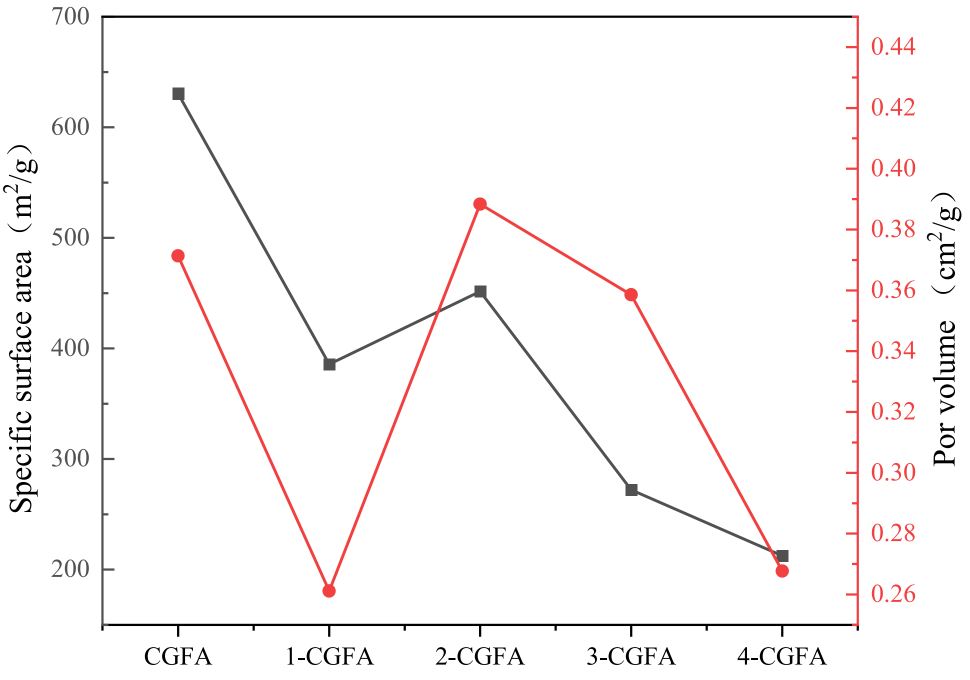


**Figure S2** Changes in specific surface area and pore volume and after CGFA modification

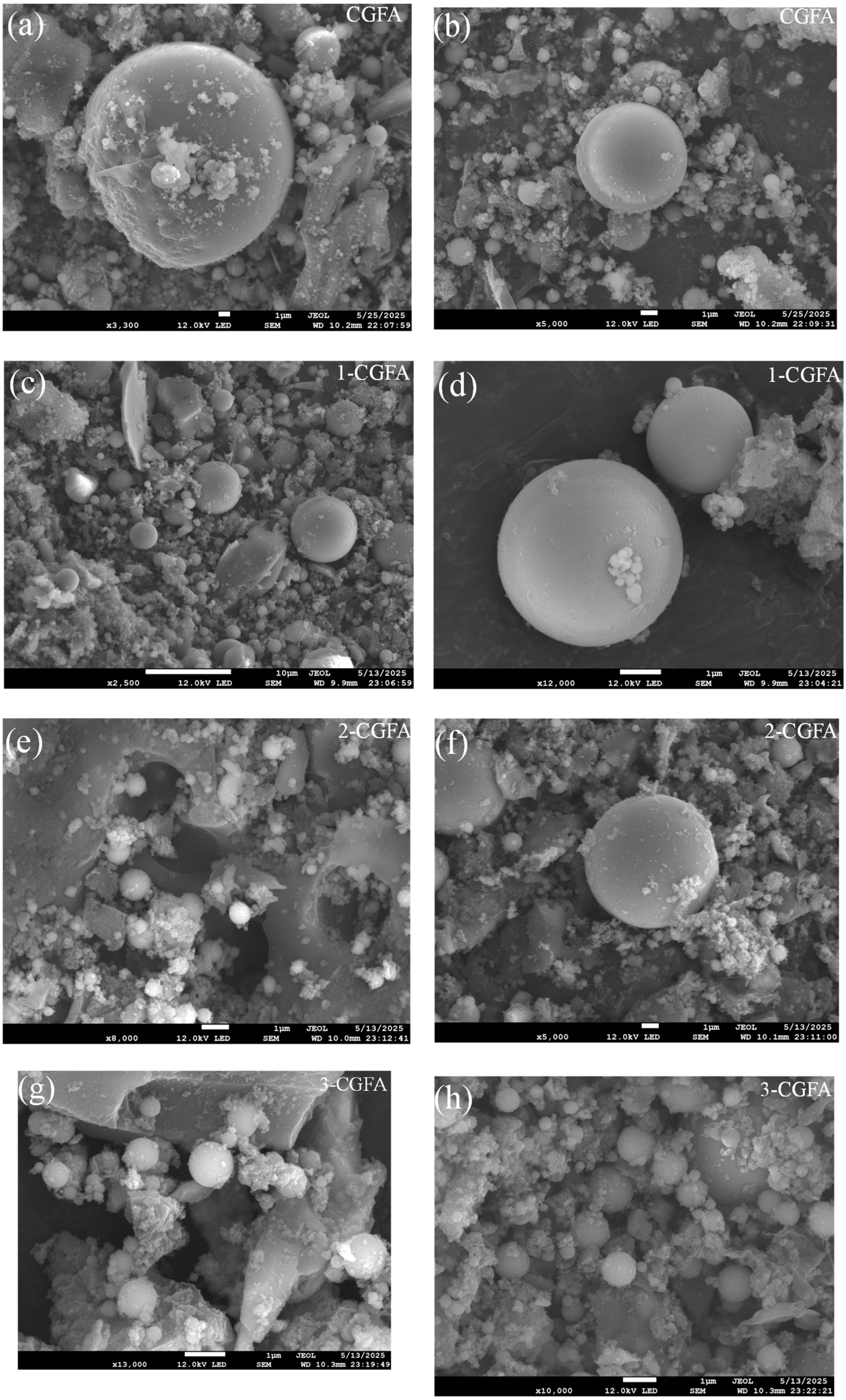
(a)
CGFA
1μm JEOL 5/25/2025
x3,300 12.0kV LED SEM WD 10.2mm 22:07:59
(b)
CGFA
1μm JEOL 5/25/2025
x5,000 12.0kV LED SEM WD 10.2mm 22:09:31
(c)
1-CGFA
10μm JEOL 5/13/2025
x2,500 12.0kV LED SEM WD 9.9mm 23:06:59
(d)
1-CGFA
1μm JEOL 5/13/2025
x12,000 12.0kV LED SEM WD 9.9mm 23:04:21
(e)
2-CGFA
1μm JEOL 5/13/2025
x8,000 12.0kV LED SEM WD 10.0mm 23:12:41
(f)
2-CGFA
1μm JEOL 5/13/2025
x5,000 12.0kV LED SEM WD 10.1mm 23:11:00
(g)
3-CGFA
1μm JEOL 5/13/2025
x13,000 12.0kV LED SEM WD 10.3mm 23:19:49
(h)
3-CGFA
1μm JEOL 5/13/2025
x10,000 12.0kV LED SEM WD 10.3mm 23:22:21

**Figure S3** SEM images

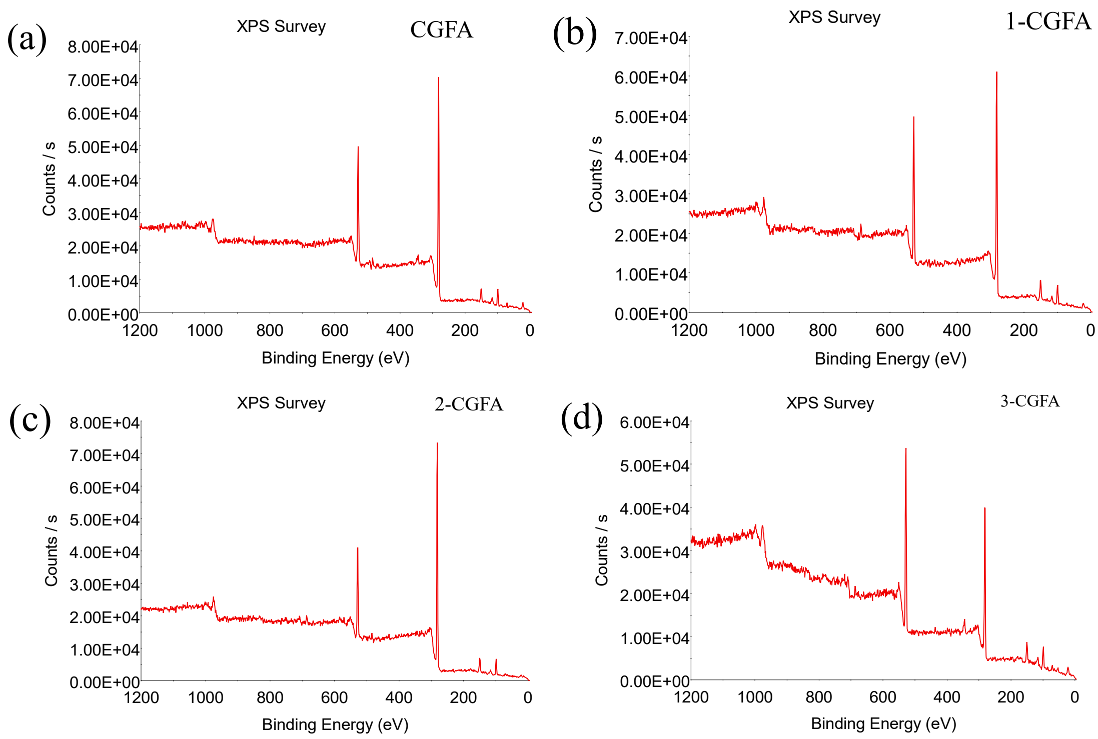


**Figure S4** XPS Survery
